# Subclinical interstitial lung damage in workers exposed to indium compounds

**DOI:** 10.1186/2052-4374-25-24

**Published:** 2013-10-21

**Authors:** Sungyeul Choi, Yong-Lim Won, Dohyung Kim, Gwang-Yong Yi, Jai-Soung Park, Eun-A Kim

**Affiliations:** 1 Occupational Safety and Health Research Institute, Korea Occupational Safety and Health Agency, Incheon, Republic of Korea; 2 Department of Radiology, Soonchunhyang University Bucheon Hospital, Bucheon, Republic of Korea

**Keywords:** Indium, Interstitial lung disease, Occupational exposure

## Abstract

**Objectives:**

The present study was designed to determine whether there is a relationship between indium compound exposure and interstitial lung damage in workers employed at indium tin oxide manufacturing and reclaiming factories in Korea.

**Methods:**

In 2012, we conducted a study for the prevention of indium induced lung damage in Korea and identified 78 workers who had serum indium or Krebs von den Lungen-6 (KL-6) levels that were higher than the reference values set in Japan (3 μg/L and 500 U/mL, respectively). Thirty-four of the 78 workers underwent chest high-resolution computed tomography (HRCT), and their data were used for statistical analysis.

**Results:**

Geometric means (geometric standard deviations) for serum indium, KL-6, and surfactant protein D (SP-D) were 10.9 (6.65) μg/L, 859.0 (1.85) U/mL, and 179.27 (1.81) ng/mL, respectively. HRCT showed intralobular interstitial thickening in 9 workers. A dose–response trend was statistically significant for blood KL-6 levels. All workers who had indium levels ≥50 μg/L had KL-6 levels that exceeded the reference values. However, dose–response trends for blood SP-D levels, KL-6 levels, SP-D levels, and interstitial changes on the HRCT scans were not significantly different.

**Conclusions:**

Our findings suggest that interstitial lung changes could be present in workers with indium exposure. Further studies are required and health risk information regarding indium exposure should be communicated to workers and employers in industries where indium compounds are used to prevent indium induced lung damage in Korea.

## Introduction

Although pneumoconiosis is still the best-known occupational chronic lower airway disease, industrial developments in recent years have introduced new manufacturing materials and production methods, which have been identified as new causes of occupational interstitial lung disease. Nylon flock and diacetyl used in the flavor industry as well as indium compounds were shown to have an association with lower airway diseases [1].

Hardly soluble indium compounds such as indium oxide, indium zinc oxide, and indium tin oxide are used in the manufacturing of electrically conductive film for liquid crystal display (LCD) panels.

Since the first report of a case of indium induced interstitial lung disease in 2003, [2] 7 other cases of indium induced interstitial lung disease were identified in Japan. In addition, 2 cases of pulmonary alveolar proteinosis (PAP) were reported in workers employed at an indium processing facility in the United States, and 1 case of PAP was reported in an worker exposed to indium in China [3-5]. Interstitial and emphysematous lung damage in workers who had been exposed to indium compounds are called “Indium Lung” [6]. Ten cases of indium lung disease as well as epidemiological studies suggest that hardly soluble indium compounds cause interstitial lung disease in workers who handle indium compounds [6-9].

Animal experiments showed that intratracheal instillation or inhalation of indium compounds result in pulmonary fibrosis [10-13]. The United States National Toxicology Program (NTP) conducted a 2-year long inhalation study of indium phosphide (InP) in mice and rats and concluded that there was clear evidence that InP is carcinogenic [14]. In 2006, the International Agency For Research On Cancer (IARC) classified InP as a probable carcinogenic agent in humans (Group 2A) [15]. Despite evidence of the harmful respiratory effects of indium compounds, there has not yet been any studies that investigated the incidence of indium induced interstitial lung disease in Korea. Therefore, this study was performed to assess whether there is a relationship between exposure to indium compounds and interstitial lung damage in workers exposed to indium compounds in Korea.

## Materials and methods

### Study subjects

This study was approved by the ethics committee of Occupational Safety and Health Research Institute (OSHRI), Korea Occupational Safety and Health Agency (KOSHA), Incheon, Korea and conducted in accordance with the declaration of Helsinki.

In total, 301 workers who handled indium compounds in the course of their work, volunteered for the study for the prevention of indium lung disease in Korea at 2012 conducted by OSHRI of KOSHA. The serum indium or Krebs von den Lungen-6 (KL-6) concentrations of 78 workers exceeded the Japanese reference values (3 μg/L and 500 U/mL, respectively) [16]. Among them, 34 workers (43.6%) agreed to undergo chest high-resolution computed tomography (HRCT), and the data of these workers were used for our statistical analysis. Health check items included completion of the OSHRI-developed questionnaire with past medical, job, and smoking histories as well as chest symptoms and signs, chest physical examinations, and spirometry as well as serum indium, KL-6, and surfactant protein D (SP-D) levels. SP-D was not used screening health check item to determine for Chest HRCT. Because Usefulness of SP-D in diagnosis of indium induced interstitial lung disease was not as good as KL-6 [6-8], and it had been listed one of secondary health check items for prevention of indium lung disease in Japan.

An occupational physician interviewed each of the workers and confirmed the accuracy of the information on the questionnaires.

### Spirometry

Spirometry was performed using PC-10 spirometer (CHEST M.I., Inc., Tokyo, Japan) to get forced vital capacity (FVC) and forced expiratory volume 1 second/forced vital capacity (FEV1/FVC). In the sitting position, the tests were repeated until obtaining appropriate and reproducible results at least three times, and then the best results were selected. All results were confirmed by physician.

### Serum KL-6 and SP-D

KL-6 is a mucin-like high molecular glycoprotein, which is strongly expressed on alveolar type II pneumocyte [17]. Serum KL-6 has been known one of sensitive biomarker for interstitial lung diseases such as idiopathic pulmonary fibrosis, radiation pneumonitis, connective tissue disease-associated interstitial lung disease, lung involvement of sarcoidosis, and pulmonary alveolar proteinosis [18,19].

SP-D is produced and secreted by type II pneumocyte. It is also elevated in patient with inflammatory lung diseases, including idiopathic pulmonary fibrosis and pulmonary alveolar proteinosis [20,21].

Serum KL-6 levels were measured by enzyme-linked immunosorbent assay (ELISA) kit (EIDIA Co., Ltd., Tokyo, Japan). And the concentration of serum SP-D was measured by human surfactant protein D ELISA kit (BioVendor, Brno, Czech). The methods and steps for analysis of KL-6 and SP-D were followed by guidelines presented by manufacturers. All samples for KL-6 and SP-D were analyzed twice, and mean values were reported and used for statistical analysis.

### HRCT scanning and radiologic evaluation

Chest HRCT was performed at the 3 hospitals near the factories when serum indium or KL-6 levels exceeded the Japanese reference values. And then all HRCT films were reviewed by a chest radiology specialist, and the reports were discussed with occupational physician.

All of the subjects underwent volumetric thin section HRCT scan. Patients were scanned caudocranially in one breath hold; 1 mm collimation was used at a table feed of 6 mm/0.75 s scanner rotation (8 mm/s) at 120 kV and 140 mA. Scanning was performed from the lung bases toward the apices. The volumetric axial images with 1 mm thickness and the 1 mm intervals were reconstructed with a high spatial frequency algorithm on supine and prone position. The images were viewed at window levels of −700 HU for analysis of thin section HRCT features. All images were displayed at the lung window setting using a PACS (picture archiving and communication system) workstation (Deja-View, Dongeun Information technology).

The thin section HRCT scans were evaluated for the presence and/or extent of the following features to evaluate indium induced interstitial lung damage:

(1) interlobular or intralobular interstitial thickening; (2) area of ground glass opacity (GGO); (3) area of emphysema. These findings were defined according to the glossary of terms recommended by the Fleischner Society [22].

The lung was divided into six zones (upper, middle, and lower, right and left) by one third and two third of the vertical distance between the lung apices and the domes of the diaphragm. Each of these zones was evaluated and scored separately for the presence and/or extent of the features on the thin section HRCT scan. Interlobular and intralobular interstitial thickening were scored in each of six zones according to the subjective score of the involved area to cross sectional area (0  =  no involvement; 1  =  minimal involvement; 2  =  moderate involvement; 3 = severe involvement) as described in other studies [23,24]. The scores of the six zones were summated as the total scores for Interlobular and intralobular interstitial thickening from 0 to 18. The remaining features of the thin section HRCT scan (ground glass opacity and emphysema) were evaluated in same scoring system for interlobular or intralobular interstitial thickening. They were expressed as a semi-quantitative scale of grade (score of interlobular and intralobular interstitial thickening + score of ground glass opacity + score of emphysema) as follows: grade 0, <1; grade 1, 1–5; grade 2, 6–10; grade 3, 11–15; grade 4, >16.

Cases of grade 1 or more were regarded positive interstitial changes, grade 0 or other suspected cases were regarded negative.

### Statistical analysis

We assessed whether continuous data were normally distributed, and data sets that were not normally distributed were log-transformed before analysis. Mean values of variables were calculated and compared with *t*-tests or ANOVA, where appropriate. Prevalence data were compared using chi-square test, Fisher’s exact tests, and Mantel-Haenszel trend test.

To compare KL-6 and SP-D levels between groups with low and high serum indium levels, the 34 subjects were divided in 2 groups: subject with serum indium levels of <10 μg/L (S-in <10) and those with ≥10 μg/L (S-in ≥10) were grouped together. Serum indium level of 10 μg/L was simple cut-off value in considering of number of subjects, to divide groups low and high serum indium levels. And To analyze the dose–response relationship between serum indium levels and KL-6 levels, SP-D levels, and HRCT finding, the workers were classified into 6 groups according to their serum indium levels (group 0: less than 1.0, group 1: 1.0–4.9, group 2: 5.0–9.9, group 3: 10.0–29.9, group 4: 30.0–49.9, and group 5: ≥50.0). P values <0.05 were considered statistically significant.

## Results

### Characteristics of the study subjects

Table 1 shows the characteristics of the study subjects. Eighteen of the workers were employed in the indium tin oxide (ITO) production industry, and the remaining 16 workers were employed at recycling plants. The average age of the 34 workers was 34.7 years (range, 20–59 years): 29 were men and 5 were women. The mean duration of indium exposure for the group was 43.4 months. Fifteen were current smokers, and 16 of the 19 non-current smokers had a history of smoking. Table 2 shows the medical history of our study subjects. Thirty-two of the 34 workers did not have a history of cardiopulmonary disease, whilst 2 had a history of hypertension.

**Table 1:** Characteristics of study subjects

**Characteristics**		**Number of subjects (%)**
n		34
Age (yr): Mean (range)		34.7 (20–59)
Sex	Male	29 (85.3)
	Female	5 (14.7)
Smoking	Currant smoker	15 (44.1)
	Ex-smoker	16 (47.1)
	Never smoker	3 (8.8)
Exposure duration (mo): mean (range)		43.4 (3–120)
Workplace	ITO production	18 (52.9)
	Recycling	16 (47.1)

ITO: indium tin oxide.

**Table 2:** The medical history of the study subjects

**Past medical history**	**N**	**%**
None	32	94.1
Pulmonary tuberculosis	0	0
Pneumonia	0	0
Asthma	0	0
COPD	0	0
Hypertension	2	5.9
Diabetes mellitus	0	0
Heart disease	0	0
Total	34	100.0

*Abbreviations*: *N* number of subjects, *COPD* chronic obstructive pulmonary disorder.

### Chest symptoms and chest physical examination

The questionnaire assessed chest symptoms and included questions regarding symptoms of cough, sputum, chest discomfort, palpitation, and wheezing. Sixteen of the 34 workers did not experience any of these symptoms, whilst 18 of the workers experienced at least 1 of the 5 chest symptoms. The prevalence rate of respiratory symptoms was higher amongst current smokers than amongst non-current smokers, but statistical significance was not reached (Table 3). The chest physical examination results of all 34 workers were normal.

**Table 3:** Respiratory symptoms

	**Current smokers**		**Ex or never smoked**		**Total**		**p**
**Respiratory symptoms**	**N**	**%**	**N**	**%**	**N**	**%**	
No	5	33.3	11	57.9	16	47.1	0.1542
Yes	10	66.7	8	41.1	18	52.9	
					34		

*Abbreviations*: *N* number of subjects, *P* probability by Chi-square test, Respiratory symptoms: at least one or more symptoms of cough, sputum, chest discomfort, palpitation, and wheezing.

### Serum immunochemistry, spirometry, and chest HRCT

Table 4 shows the results of the serum immunochemistry. Geometric means (GM) and geometric standard deviations (GSD) of serum indium, KL-6, and SP-D in the workers were 10.9 (6.7) μg/L, 859.0 (1.9) U/mL, and 179.3 (1.8) ng/mL, respectively.

**Table 4:** Serum immunochemistry

**Serum immunochemistry**	**n**	**GM**	**GSD**	**Range**	**p**
Serum indium (μg/L)	34	10.9	6.7	ND-125.8	
S-in <10	16	2.5	6.2	ND-9.2	<0.0001
S-in ≥10	18	39.4	2.2	11.7-125.8	
KL-6 (U/mL)	34	859.0	1.9	274.6-2062.2	
S-in <10	16	704.4	1.8	242.6-942.7	0.0760
S-in ≥10	18	1024.6	1.8	948.0-2062.2	
SP-D (ng/mL)	34	179.3	1.8	60.0-466.1	
S-in <10	16	135.0	1.7	59.9-177.0	0.0064
S-in ≥10	18	230.7	1.7	183.4-466.1	

*Abbreviations*: *GM* geometric mean, *GSD* geometric standard deviation, *S-in* serum indium, *KL-6* Krebs von den Lungen-6, *SP-D* surfactant protein D, P probability by Student *t*-test.

The GM of KL-6 in group S-in <10 and in group S-in ≥10 were 704.4 U/mL and 1024.6 U/mL, respectively, and the GM of SP-D in group S-in <10 and S-in ≥10 was 135.0 ng/mL and 230.7 ng/mL, respectively. Although the GM of KL-6 and SP-D for subjects with S-in ≥10 were higher than that for subjects with S-in <10, only SP-D levels differed significantly between groups, whilst differences in KL-6 levels did not reach significance.

The FEV_1_/FVC ratio of all 34 workers was within the normal range of >70%, whilst the % FVC were within the normal range of >80% for all except 2 workers. These 2 workers had % FVC values of 73.4% and 78.8%, respectively, and each of them had mild restrictive patterns. Nine of the 34 workers had evidence of interstitial changes on chest HRCT (Grade 1: 3 cases, Grade 2: 4 cases, Grade 3: 2 cases). The main HRCT findings were intralobular interstitial thickening, whilst there were no workers with ground glass opacities or emphysematous changes.

**Figure 1:**
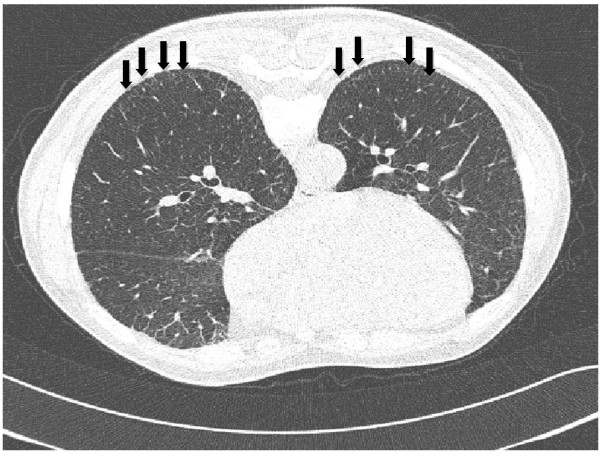
**High-resolution computed tomography scans on prone position of a worker with no history of smoking, who had been employed at an indium tin oxide target production facility for 7 years (sintering room for 4 years and indium oxide powder room for 3 years).** The image shows subpleural interlobular interstitial thickening in both lower lung fields (arrows). The patient had no history of respiratory diseases. Results of health examinations were as follows: serum indium, 50 μg/L; KL-6, 1769 U/mL; FVC, 86% predicted; FEV1/FVC, 109.6% predicted.

GM and GSD of KL-6 and SP-D, prevalence (%) for exceeding the reference values of KL-6 and SP-D, and interstitial changes on chest HRCT in each group are shown in Table 5. Dose-dependent trends for KL-6 and SP-D were statistically significant when age- and smoking-adjusted multiple regression model was applied. 5.40+ 0.1360* serum indium for KL-6 (p = 0.0055) and 5.25+ 0.1846* serum indium for SP-D (p = 0.0005). Results of Dunnett’s test revealed that compared to Group 0, the means KL-6 value of groups 4 and 5 increased significantly. Mean values of SP-D increased in patients with serum indium levels ≥10 μg/L, but statistical significance was not reached. The KL-6 levels of all 12 workers in Group 4 and 5 were above the reference value. A dose–response trend for the prevalence of SP-D, but not for the prevalence of KL-6 and interstitial changes on HRCT, was statistically significant. The prevalence of these changes on the HRCT in each group was not significantly different (0.0%–42.9%) (Figure 1).

**Table 5:** Dose–response relations between serum indium and KL-6, SP-D, and interstitial changes on chest HRCT

	**Serum indium (μg/L)**			**KL-6 (U/mL)**		**SP-D (ng/mL)**		**Prevalence (%)**		
**Group**	**Range**	**GM**	**n**	**GM**	**GSD**	**GM**	**GSD**	**KL-6 (>500 U/mL)**	**SP-D (>110 ng/mL)**	**HRCT-I**
0	<1.0	0.2	3	586.1	1.3	120.9	2.9	100.0	33.3	0.0
1	1.0-4.9	3.4	7	509.6	1.7	143.8	1.5	42.9	71.4	14.3
2	5.0-9.9	7.4	6	1126.9	1.6	132.5	1.6	83.3	66.7	33.3
3	10.0-29.9	15.5	6	486.3	1.4	157.6	1.6	50.0	83.3	16.7
4	30.0-49.9	43.8	5	1539.4^*^	1.5	252.6	1.9	100.0	80.0	40.0
5	≧50	81.5	7	1451.1^*^	1.1	299.8	1.3	100.0	100.0	42.7
p for trend				0.0055		0.0005		0.1057	0.0353	0.1198

*Abbreviations*: *GM* geometric mean, *GSD* geometric standard deviation, *KL-6* Krebs von den Lungen-6, *SP-D* surfactant protein D, *HRCT-I* interstitial changes on HRCT, Prevalence (%): % of workers exceeding each reference values and % of workers with interstitial changes on HRCT, p for trend: age- and smoking-adjusted multiple regression model for GM and GSD of KL-6 and SP-D, Mantel-Haenszel trend for prevalence data, * P < 0.05 by Dunnett’s test.

## Discussion

In this study we did not clarify the dose-effect and dose–response relations between indium compound exposure and interstitial lung changes on HRCT. However, we found significant dose-effect and dose–response relations between serum indium levels and GM of KL-6 and the prevalence of exceeding reference value of SP-D in workers exposed to indium, despite of the insignificant difference of the prevalence of exceeding reference value of KL-6 and HRCT. Although we did not include non-exposed individuals in our study, our results clearly show that the GM of KL-6 and SP-D levels were higher in workers with serum indium levels ≥10 ug/L than in those with serum indium levels <10 ug/L.

Our finding showed similar results the studies of Japan [7-9] reported that workers exposed to indium compounds had significantly higher serum indium, KL-6, and SP-D levels than non-exposed workers. In addition, serum indium level was positively correlated with KL-6 and SP-D levels. The rates of exceeding reference value of serum indium, KL-6, and SP-D were statistically high in exposed workers. However, the prevalence of interstitial thickening on HRCT identified interstitial lung- and emphysematous changes in exposed workers were not significantly different from that in non-exposed workers. These studies reported dose-effect and dose–response relationships between serum indium and KL-6, SP-D, and HRCT, and found no significant differences in KL-6 and SP-D levels between current smokers and non-current smokers.

Insignificant relationship of KL-6 and HRCT in this study might be influenced by the limited number of the study subjects. Because relatively small number of the workers having high level of KL-6 had volunteered for HRCT (43.6%) when compared with that in previous studies, the participants might be influenced by selection bias, and this could affect to the unclear dose-effect and dose–response relations for all parameters of this study. This study was conducted for screening interstitial lung disease and gathering basic health information in indium exposed workers, so interstitial lung damages were not confirmed pathologically. There were no statistical differences in the prevalence of KL-6, SP-D, and interstitial changes as seen on HRCT between current smokers and non-current smokers (data not shown).

The pathogenesis of indium related lung disorder is not understood yet. According to the 2001 NTP report, [14] the incidence of adenoma and lung cancer increased in laboratory rats and mice exposed to InP, and the extent and severity of inflammatory lesions were much greater and included atypical hyperplasia, chronic inflammation, alveolar epithelial hyperplasia and metaplasia, alveolar proteinosis, and interstitial fibrosis. In the same manner, the reported cases related with indium found various clinical syndromes. Interstitial thickening and emphysematous changes in the lungs, as seen on HRCT, were evident in 7 patients who worked in grinding process of indium tin oxide target manufacturing factory of indium lung disease in Japan. However, 2 patients worked in reclaiming facility in the United States and 1 patient in China who was exposed to indium compound in display production.

Interstitial lung fibrosis cases in Japan showed intraalveolar filling of cholesterol crystal, which is pathological features of pulmonary alveolar proteinosis. The pulmonary lesion of Japanese cases were not only confined in intraalveolar space, but also infiltrated interstitium. Thus, Japanese cases may represent pulmonary alveolar proteinosis with interstitial involvement [4].

The pathogenesis of indium toxicity is not clear. Therefore, the reasons underlying the different pathologic findings after indium exposure remain unclear. One hypothesis that could explain the pathogenesis of indium lung disease is the notion that indium induces a “foreign body reaction” [4,5]. This hypothesis suggests that when indium compounds are inhaled into the lungs, a foreign body reaction and macrophage dysfunction in the lungs are induced, leading accumulation of intraalveolar lipoproteinaceous material and development of cholesterol ester crystals with the formation of cholesterol granulomas and this results in eventual interstitial fibrosis and emphysema. However, this hypothesis does not sufficiently explain the pathologic findings of fibrotic foci in areas without cholesterol ester crystals. Other undiscovered pathogenic mechanisms for indium lung disease may exist.

Tin is a well-known cause of stannosis, benign pneumoconiosis without fibrosis, or necrosis [25,26]. Previous animal experiments [6-9,14] and an epidemiological study [8] reported that tin-free indium compounds induced lung fibrosis. It is thought that pulmonary interstitial fibrosis in workers exposed to indium tin oxide could be attributed to indium compounds.

We used serum indium levels as a biological exposure index for indium compound exposure in this study. Based on the 2001 NTP report, [14] serum indium levels increased proportionally with an increase in the duration of exposure, although serum indium levels were low relative to indium concentrations in the lungs. And the decrease in serum indium levels was slower than indium clearance in the lungs. KL-6 and SP-D were used as an index to evaluate pulmonary interstitial damages. But biological half-life of indium compound was not been defined clearly.

Workers enrolled in our study were from different indium processing facilities in different parts of Korea, and it was therefore not possible for us to perform chest HRCT in 1 hospital only. Therefore, the quality of chest HRCT may not have been uniform.

## Conclusion

In Korea, the demand for indium compounds increased with the expansion of the display manufacturing industry, but the extent to which indium is used in this country remains unclear. In 2011, the domestic demand for indium in Korea was estimated to be 950 tons [27]. This estimate was similar to the amount used in Japan in 2006 [6]. According to this estimation, Korea is one of the foremost consumers of indium in the world. Although our study had some limitations, to our knowledge, it is the first investigation of indium induced interstitial lung damage in Korea, and our results are meaningful as an initial step in the identification and prevention of indium induced lung damage in Korea. Nakano *et al*. [7] investigated serum indium, KL-6, and SP-D levels before and after engineering control, which included the use of appropriate respiratory protective devices, improvement of workplace ventilation and found that serum indium, KL-6, and SP-D levels decreased after these improvements. Further studies are required to clarify pathogenesis of indium induced interstitial lung damage. Health risk information regarding indium exposure should be communicated to workers and employers in industries where indium compounds are used to prevent indium induced lung damage in Korea.

## Consent

Written informed consent was obtained from all the workers for the publication of this report and any accompanying images.

## Competing interests

The authors declare that they have no competing interests.

## Authors’ contributions

CSY and KEA conceived and designed the study. KDH, CSY, YGY and WYI were involved in conduction of the study. YGY measured the environmental exposure and WYL performed the analysis of the biological index. PJS conducted imaging study. KEA, CSY and KDH interpretation of data. All authors read and approved the final manuscript.
